# Transcriptome and Coexpression Network Analyses Reveal Hub Genes in Chinese Cabbage (*Brassica rapa* L. ssp. *pekinensis*) During Different Stages of *Plasmodiophora brassicae* Infection

**DOI:** 10.3389/fpls.2021.650252

**Published:** 2021-08-10

**Authors:** Yuxiang Yuan, Liuyue Qin, Henan Su, Shuangjuan Yang, Xiaochun Wei, Zhiyong Wang, Yanyan Zhao, Lin Li, Honglei Liu, Baoming Tian, Xiaowei Zhang

**Affiliations:** ^1^Institute of Horticulture, Henan Academy of Agricultural Sciences, Graduate T&R Base of Zhengzhou University, Zhengzhou, China; ^2^School of Life Sciences, Zhengzhou University, Zhengzhou, China

**Keywords:** Chinese cabbage, *Plasmodiophora brassicae*, clubroot, hub genes, transcriptome, coexpression network analysis

## Abstract

Clubroot, caused by the soil-borne protist *Plasmodiophora brassicae*, is one of the most destructive diseases of Chinese cabbage worldwide. However, the clubroot resistance mechanisms remain unclear. In this study, in both clubroot-resistant (DH40R) and clubroot-susceptible (DH199S) Chinese cabbage lines, the primary (root hair infection) and secondary (cortical infection) infection stages started 2 and 5 days after inoculation (dai), respectively. With the extension of the infection time, cortical infection was blocked and complete *P. brassica* resistance was observed in DH40R, while disease scales of 1, 2, and 3 were observed at 8, 13, and 22 dai in DH199S. Transcriptome analysis at 0, 2, 5, 8, 13, and 22 dai identified 5,750 relative DEGs (rDEGs) between DH40R and DH199S. The results indicated that genes associated with auxin, PR, disease resistance proteins, oxidative stress, and *WRKY* and *MYB* transcription factors were involved in clubroot resistance regulation. In addition, weighted gene coexpression network analysis (WGCNA) identified three of the modules whose functions were highly associated with clubroot-resistant, including ten hub genes related to clubroot resistance (*ARF2*, *EDR1*, *LOX4*, *NHL3*, *NHL13*, *NAC29*, two *AOP1*, *EARLI 1*, and *POD56*). These results provide valuable information for better understanding the molecular regulatory mechanism of Chinese cabbage clubroot resistance.

## Introduction

Chinese cabbage (*Brassica rapa* L. ssp. *pekinensis*) is an economically important cruciferous vegetable worldwide (Jia et al., 2017). Clubroot, caused by the soil-borne protist plant pathogen *Plasmodiophora brassicae*, is considered a destructive disease of *Brassica* crops. In recent years, the incidence rate and severity of clubroot have increased with intensified Chinese cabbage breeding and cultivation, resulting in great losses in Chinese cabbage quality and yields in China and worldwide (Irani et al., 2018).

The life cycle of *P. brassicae* consists of primary infection (root hair infection) and secondary infection (cortical infection) stages. During root hair infection, primary zoospores are released from the resting spores to infect the root hairs and then divide to form the secondary sporangium. During cortical infection, the released secondary zoospores directly infect cortical cells, where the secondary plasma mass forms. Finally, secondary plasmodia divide to form numerous resting spores, which are released into the soil and become the initial infection source in the future (McDonald et al., 2014). Once contaminated, these *P. brassicae* spores are long lived and resistant to severe environmental conditions, making the disease difficult to control in the field (Kageyama and Asano, 2009). Therefore, studies on the molecular basis of clubroot contamination in cruciferous plants are urgently needed.

Many disease resistance genes, including *CRa* or *CRb*, *CRc*, *CRd*, *CRk*, *CRs*, *Crr1*, *Crr2*, *Crr3*, *Crr4*, *PbBa3.1*, and *PbBa3.3* have been identified from different genetic resources and are associated with distinct *P. brassicae* pathotypes (Hirai et al., 2003; Suwabe et al., 2003; Piao et al., 2004; Suwabe et al., 2006; Sakamoto et al., 2008; Schönbach et al., 2013; Pang et al., 2018; Laila et al., 2019). Despite the discovery of so many resistance sites, only three *CR* genes, including *CRa* (Ueno et al., 2012), *Crr1a* (Hatakeyama et al., 2013) and *CRb* (Hatakeyama et al., 2016), have been cloned. *CRa* and *Crr1a* are known to encode Toll-interleukin receptor (TIR)-nucleotide binding site (NBS)-leucine-rich repeat (LRR) proteins, and it has been reported that *CRb* and *CRa* are the same gene (Hatakeyama et al., 2016). Although clubroot resistance (*CR*) genes have been identified, mainly in *Brassica rapa*, the detailed defense response mechanism of *CR* genes remains unclear.

The analysis of dynamic changes in gene expression is a useful way to explore the molecular basis of clubroot resistance in *Brassica* species. Many studies conducted to date have focused on molecular mechanisms aided by “-omics” approaches, such as RNA-seq. Mei et al. (2019) reported great differences between two rapeseed genotypes, especially in the activation of signaling networks, the production of reactive oxygen species (ROS) and the programmed cell death (PCD) response to *P. brassicae* (Mei et al., 2019). Chu et al. (2014) found that the genes involved in the jasmonate (JA) and ethylene (ET) signaling and metabolic pathways and the defensive deposition of callose were significantly upregulated in clubroot-resistant plants compared to their levels in susceptible lines at 15 days after infection (Chu et al., 2014). Additionally, based on a transcriptome analysis of *B. rapa* during the early stages of infection by *P. brassicae*, Chen et al. (2016) confirmed that genes associated with pathogen-associated molecular patterns, calcium ion influx, hormone signaling, pathogenesis-related (PR) pathways, transcription factors, effector receptors and cell wall modification played important roles during early infection stages (Chen et al., 2016). Above all, the identified differentially expressed genes (DEGs) are related to hormone signaling, cell wall modification, NBS-LRR proteins, Ca^2+^ signaling, defense-related callose deposition, chitin metabolism, and the PR in response to *P. brassicae* (Jubault et al., 2008; Schuller et al., 2014; Zhang et al., 2016).

These previous studies mostly focused on understanding the defense mechanisms involved in resistance to clubroot during the primary or secondary stages of infection, and differences have been identified between genotypes and sampling time points during the pathogen response after *P. brassicae* infection. However, few transcriptomic studies have focused on the regulatory interaction network involved in resistance to clubroot during all stages of infection by *P. brassicae* in Chinese cabbage. In this study, comparative cytological observations and transcriptome analysis were applied to the roots of two genotypes (the clubroot-resistant line DH40R and the susceptible line DH199S) after *P. brassicae* infection during both the primary and secondary stages to identify the key pathways and genes that could be involved in clubroot resistance.

## Materials and Methods

### Plant Materials and *P. brassicae* Inoculation

Two Chinese cabbage DH lines (DH40R: resistant, R-line, *B. rapa*, 2n = 2x = 20; and DH199S: susceptible, S-line, *B. rapa*, 2n = 2x = 20) that show contrasting performance regarding resistance to *P. brassicae* and have been subjected to resistance identification in different years were used in the present study. These two lines were developed by isolated microspore culture at the Institute of Horticulture, Henan Academy of Agricultural Sciences. The seeds of the R- and S-lines were germinated on wet filter paper for 3 days, transferred to plastic trays with a nutrient substrate and grown under controlled conditions at 20–25°C with a photoperiod of 16 h of light and 8 h of darkness.

The pathogen used in this study was collected from an infected field (IF) at Xinye, Henan, China, where the pathogen was reported to be pathotype 4 based on Williams classification (Wei et al., 2016). The methods for obtaining *P. brassicae* suspensions were as described in a previous study (Galindo-Gonzalez et al., 2020), and the resting spore concentration in the suspension was adjusted to a density of 10^7^ spores/mL with sterile distilled (SD) water according to measurement using a haemocytometer. After being transferred to plastic trays, 20-days-old seedlings were individually injected at the stem bottom with 4 mL of the spore suspension (1.0 × 10^7^ spores/mL), and the soil was kept moist throughout the treatment period.

### Cytological Observation of the Infection Process

Every 24 h after inoculation, a microscope (Leica Microsystems CMS GmbH Ernst-Leitz-Str, Germany) was used to monitor the dynamic *P. brassicae* infection process in the roots of the two Chinese cabbage genotypes until secondary infection was confirmed (Mei et al., 2019). One or two plants were removed from the soil every 2 days to observe the development of the disease.

At 0, 2, 5, and 8 dai, the occurrence and development of root hair infection by *P. brassicae* in Chinese cabbage were observed, and the detailed steps are described below. The roots were washed with flowing water, and a segment was cut from the lateral roots under each treatment at each time point. The segments were stained with FAA for a minimum of 24 h and with 0.5% phloxine B for 3 h at room temperature, after which they were covered with a coverslip and viewed with a microscope (Xu et al., 2018).

The development of cortex infection by *P. brassicae* was examined at 13 and 22 dai. The roots were washed with flowing water, and a segment was cut and fixed in FAA for 24 h. After routine dehydration and wax leaching, the samples were embedded in paraffin and sliced into 4-μm-thick sections. Finally, the sections were observed and imaged under an optical microscope (Xu et al., 2018).

In addition, the ratio of infected root hairs was investigated in 100 root hairs per plant between 2 and 13 dai, and the average number of infected cortices in the roots was counted from three microscopic fields per plant at 5 (the beginning of infection in the cortical cells), 8 (serious infection in the cortical cells, disease scale of 1 in DH199S), 13 (disease scale of 2 in DH199S) and 22 dai (disease scale of 3 in DH199S). Plants of the disease-resistant (DH40R) and disease-susceptible (DH199S) lines were selected to analyze transcriptional changes after inoculation at each time point (0, 2, 5, 8, 13, and 22 dai). Under each treatment, sampled roots were pooled from at least three individual plants, and three biological replicates were performed at each time point. The roots were cleaned with distilled water, wrapped in tin foil, quickly frozen in liquid nitrogen and stored at –80°C. To verify successful infection after the inoculation of *P. brassicae*, 40 plants of each genotype were evaluated for resistance identification until 35 days after inoculation.

### RNA Extraction and Construction of cDNA Sequencing Library

Total RNA from 36 samples was extracted using the RNAprep Pure Plant Kit (Tiangen, Beijing, China) following the manufacturer’s instructions. RNA purity was checked using a KaiaoK5500^®^ Spectrophotometer (Kaiao, Beijing, China). RNA integrity and concentrations were assessed using the RNA Nano 6000 Assay Kit and a Bioanalyzer 2,100 system (Agilent Technologies, CA, United States). A total of 2 μg of RNA per sample was used as the input material for RNA sample preparation. First-strand cDNA was synthesized using random hexamer primers and RNase H. Second-strand cDNA synthesis was subsequently performed using buffer, dNTPs, DNA polymerase I and RNase H. The library fragments were purified with QiaQuick PCR kits and eluted with EB buffer. Then, terminal repair was performed, the desired products were retrieved, PCR was performed, and the library was completed.

### RNA Sequencing and Data Preprocessing

All 36 libraries were sequenced on the Illumina HiSeq platform, and 150 bp paired-end raw reads were generated. Before assembly, various quality control measures were applied to the raw data. High-quality sequences (clean data) were obtained from the original offline data sequences by removing reads containing adapters and low-quality reads. All subsequent analyses are based on the filtered clean data. Bowtie2 v2.2.3 was used to build the genome index, and clean data were then aligned to the reference genome using HISAT2 v2.1.0 (Langmead et al., 2009; Kim et al., 2015). The sequencing reads were mapped to the *B. rapa* genome^1^, and all of the raw reads were deposited in the NCBI Short Read Archive (SRA) database under accession number PRJNA692311.

### Analysis of Differential Gene Expression and Gene Annotation

DESeq2 software was employed to calculate the expression of three biological replicates between two samples (Wang et al., 2010). Linear regression was used to estimate the gene expression intensity in each sample, and the Wald test was used to calculate non-differential gene expression in the two sets of samples. The genes with a | log_2_ fold change| ≥ 1 and FDR-adjusted *p*-value (*q*-value) < 0.05 were selected as DEGs. All expressed genes were functionally annotated by using the NCBI non-redundant protein database (NR) protein database, UniProt database, Cluster of Orthologous Groups (COG) database, Gene Ontology (GO) database and Kyoto Encyclopedia of Genes and Genomes (KEGG) database. The FPKM of all expressed genes are listed in Supplementary Table 1.

In this study, to better distinguish DEGs, the concept of relative period differentially expressed genes (rDEGs) was proposed; that is, when a gene was identified as a DEG between the R- and S-lines, but also identified as a DEG in the R-line compared with the R-previous period line at the corresponding time points, the gene was defined as a rDEG (Li et al., 2020). GO enrichment analysis, KEGG pathway analysis and heatmap generation were performed using TBtools (Chen C. et al., 2018).

### Construction of Gene Coexpression Networks and Prediction of Hub Genes

To comprehensively and systematically investigate the gene regulatory network and identify the specific genes that are highly associated with clubroot resistance, a WGCNA was performed. Coexpression networks were constructed on the basis of pairwise correlations of gene expression across all samples. The coexpression modules were defined as clusters of highly interconnected genes, and these genes within the same cluster exhibited high correlation coefficients. A module consisted of a cluster of highly interconnected genes showing similar expression changes related to a certain physiological process (Du et al., 2017). The coexpression network analysis was conducted using the WGCNA version package in R software (Langfelder and Horvath, 2008). Module identification was implemented after merging modules whose expression profiles were similar with a merge cutHeight of 0.5. The interaction network of hub genes in a module was visualized using Cytoscape 3.8.0.

### Validation of RNA-Seq Data by Quantitative Real-Time-Polymerase Chain Reaction

The transcript levels of DEGs were identified using quantitative real-time polymerase chain reaction (qRT-PCR). A TaKaRa MiniBEST Plant RNA Extraction Kit (TaKaRa, Dalian, Liaoning, China) was used to extract the total RNA following the manufacturer’s protocol.^2^ A RevertAid First Strand cDNA Synthesis Kit (TaKaRa) was used to reverse transcribe RNA to obtain cDNA. qRT-PCR was conducted as previously described (Yang et al., 2021). GAPDH was used as an internal reference gene for Chinese cabbage (Su et al., 2015). The amplification efficiency of the primers were detected by qRT-PCR, all primers used for qRT-PCR are listed in Supplementary Table 2. Each qRT-PCR experiment was performed in three replicates and the resultant mean value was used for qRT-PCR analysis. The relative expression levels of the target genes were calculated by using the 2^–△△Ct^ method (Schmittgen and Livak, 2008).

## Results

### Identification of Disease Resistance and Morphological Changes After Inoculation in DH40R and DH199S

**FIGURE 1:**
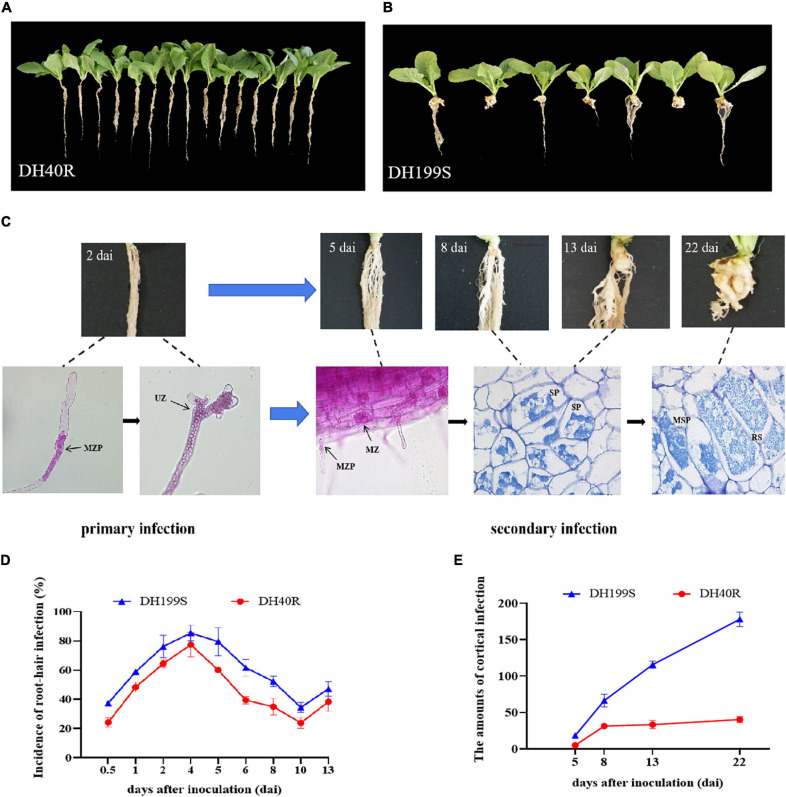
Morphology analysis and the dynamic changes of root-hair infection and cortical infection between DH40R and DH199S infected by *P. brassicae*. **(A)** DH40R; **(B)** DH199S. **(A,B)** 35 dai; **(C)** Infection dynamics in the root hairs of DH199S. **(D,E)** The dynamic changes of root-hair infection and cortical infection in DH40R and DH199S. MZP, multinucleate zoosporangia plasmodium; UZ, uninucleate zoosporangia; MZ, multinucleate zoosporangia; SP, secondary plasmodium; MSP, multinucleate secondary plasmodium; RS, resting spore.

The disease index (DI) was investigated 35 days after inoculation (dai) with *P. brassicae* in the two Chinese cabbage DH lines. DH40R plants infected with *P. brassicae* showed no gall formation (Figure 1B) with a mean DI of 0, while DH199S plants were susceptible with a mean DI of 95 (Supplementary Table 3), and they showed severe galls on both the lateral roots and main roots (Figure 1A).

Phloxine B-stained root hairs were microscopically examined to follow the *P. brassicae* infection process at 0, 2, 5, and 8 dai in DH40R and DH199S. At 0 dai (before infection), the root hairs of DH40R and DH199S were empty and showed no signs of infection (Supplementary Figure 1); at 2 dai, the root hairs of DH199S and DH40R were filled with primary plasmodia (Figure 1C); at 5 dai, a small amount of *P. brassicae* secondary plasmodia was observed in the cortical cells of DH40R (Supplementary Figure 1), while abundant secondary plasmodia appeared in the cortical cells of DH199S (Figure 1C); at 8 dai, the number of secondary plasmodia in cortical cells was higher in DH199S than in DH40R; at 13 dai, there were filled with young secondary plasmodia in DH199S (Figure 1C); and at 22 dai, different stages of plasmodia development could be observed inside the cortical cells of DH199S, including secondary plasmodia and resting spores, with secondary plasmodia causing cell hypertrophy, expansion and gall formation in the root tissues of DH199S (Figure 1C), while in contrast, the resistant roots of DH40R grew normally during this stage (Figure 1B).

In summary, root hair infection and cortical infection started in both DH40R and DH199S on 2 dai and 5 dai, respectively. However, the root hair and cortical infection rates were higher in DH199S than in DH40R during the entire infection process (Figure 1D and Supplementary Table 3). Starting on 5 dai, the cortical infection rate of DH199S continued to increase, whereas that of DH40R remained basically unchanged from 8 dai onward (Figure 1E and Supplementary Table 3). In the two genotypes, 8 dai was an important time point in the gradual development of different resistance levels. The results suggested that DH40R and DH199S were successfully infected and could be reliably used for further study.

### The Data Analysis of RNA-Seq

To study the different molecular mechanisms underlying resistance in the two lines after *P. brassicae* infection, root samples were collected, and RNA was extracted from three biological replicates from 6 time points (0, 2, 5, 8, 13, and 22 dai) for both DH40R and DH199S. A total of 36 cDNA libraries were constructed, and approximately 25.6–31.9 million clean reads were generated from the libraries through RNA-seq. The percentage of clean read rates was higher than 92% in all cases, and the Q30 base percentages were all above 93%, indicating that the accuracy and quality of the data obtained were sufficient for further analysis (Supplementary Table 4). In general, 63∼94% of the high-quality reads in each sample could be mapped to the 3.0 version of the Chinese cabbage genome.

### Identification of Differentially Expressed Genes

**FIGURE 2:**
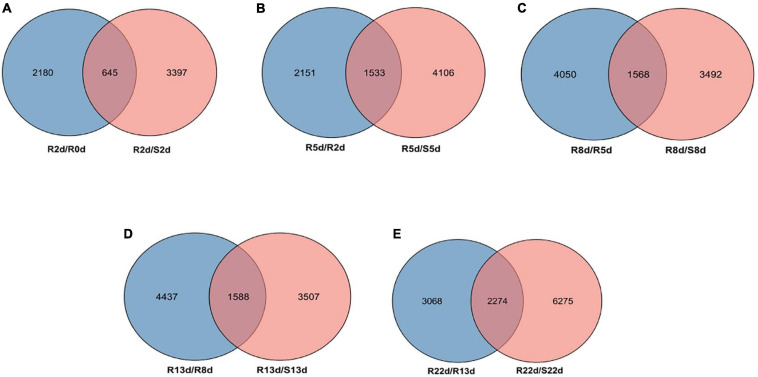
The differentially expressed genes (DEGs) profiles. **(A)** Venn diagram of DEGs between the R2 vs. R0 and R2 vs. S2. **(B)** Venn diagram of DEGs between the R5 vs. R2 and R5 vs. S5. **(C)** Venn diagram of DEGs between the R8 vs. R5 and R8 vs. S8. **(D)** Venn diagram of DEGs between the R13 vs. R8 and R13 vs. S13. **(E)** Venn diagram of DEGs between the R22 vs. R13 and R22 vs. S22.

According to the criteria of a | log_2_ fold change| ≥ 1 and *q*-value < 0.05, a total of 5,268, 4,042, 5,639, 5,060, 5,095, and 8,549 DEGs in DH40R vs. DH199S were obtained at 0, 2, 5, 8, 13, and 22 dai, respectively. In addition, we obtained 5 groups of rDEGs at 5 time points after inoculation (Figures 2A–E). In Figure 2A, there were 645 DEGs in common between DH40R-2dai vs. DH199S-2dai and DH40R-2dai vs. DH40R-0dai; in Figure 2B, there were 1,533 DEGs in common between DH40R-5dai vs. DH199S-5dai and DH40R-5dai vs. DH40R-2dai; in Figure 2C, there were 1,568 DEGs in common between DH40R-8dai vs. DH199S-8dai and DH40R-8dai vs. DH40R-5dai; in Figure 2D, there were 1,588 DEGs in common between DH40R-13dai vs. DH199S-13dai and DH40R-13dai vs. DH40R-8dai; in Figure 2E, there were 2,274 DEGs in common between DH40R-22dai vs. DH199S-22dai and DH40R-22dai vs. DH40R-13dai. Above all, a total of 5,750 rDEGs (Supplementary Table 5) were obtained after removing 1,858 duplicates in those 5 groups, and the 5,750 rDEGs were used for the next functional enrichment analysis.

### Functional Annotation of DEGs

**FIGURE 3:**
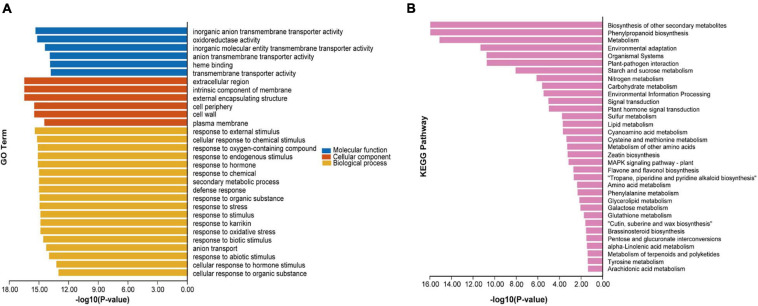
Gene ontology (GO) and Kyoto Encyclopedia of Genes and Genomes (KEGG) enrichment analysis of rDEGs. **(A)** GO function classification of 5,750 rDEGs. **(B)** KEGG pathway of 5,750 rDEGs.

GO term enrichment analysis was performed on the rDEGs to classify their gene functions according to three main GO terms: biological process (BP), cellular component (CC) and molecular function (MF). A total of 3,911 DEGs were assigned to these three GO categories. For the BP ontology, the main enriched terms were “response to stimulus” (including external, chemical, endogenous, biotic, hormone, and abiotic), “response to stress” (including oxygen-containing compounds and oxidative stress), “secondary metabolic process” and “defense response” (Figure 3A).

To better understand the biological significance of the gene functions of the identified rDEGs, these genes were mapped to reference canonical pathways in the KEGG database. A total of 33 pathways were found to be enriched, among which 24 pathways were significantly enriched (*p* < 0.05). Among the top 10 significant mapped pathways, “biosynthesis of secondary metabolites” “phenylpropanoid biosynthesis” and “metabolism” were the most significantly enriched (Figure 3B).

### Construction of Gene Coexpression Networks

**FIGURE 4:**
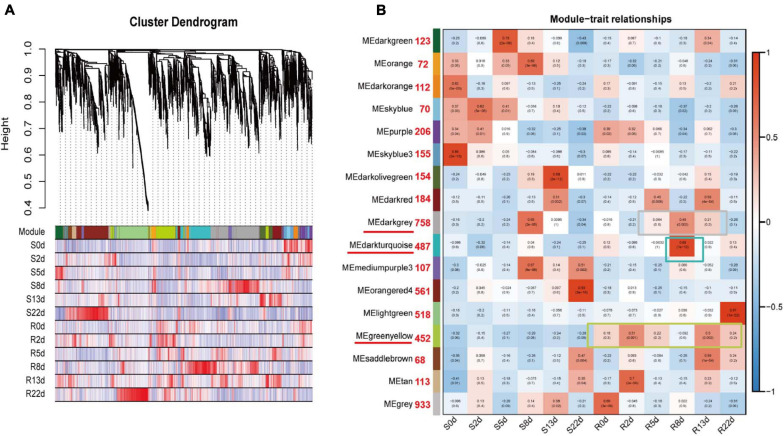
Weighted gene coexpression network analysis (WGCNA) of rDEGs in DH40R and DH199S at 0, 2, 5, 8, 13, 22 dai. **(A)** Hierarchical cluster tree showing coexpression modules identified by WGCNA. Each leaf in the tree represents one gene. **(B)** Module-sample group association analysis. Each row corresponded to a module, labeled with a color as in **(A)**, and each column corresponded to a sample group. The color of each cell at the row column intersection indicates the correlation coefficient between the module and the sample group. Those numbers next to the module represent the number of genes contained in each module.

To obtain a comprehensive understanding of genes expressed in successive developmental stages across the two genotypes after inoculation and to identify specific genes that were highly associated with resistance to *P. brassicae*, WGCNA of 5,750 rDEGs was carried out. Coexpression networks were constructed on the basis of pairwise correlations of gene expression across all samples. Modules were defined as clusters of highly interconnected genes, and genes within the same cluster exhibited high correlation coefficients. After filtering out the 677 rDEGs with low expression (FPKM < 1), 5,073 rDEGs assigned to seventeen distinct modules (marked in different colors) were included in the cluster dendrogram, in which each tree branch represented a module and each leaf in the branch represented one gene (Figure 4A).

Notably, three of the coexpression modules were composed of genes that were highly expressed in a specific sample, suggesting that they play important roles in resistance to *P. brassicae*; these modules are underlined in red and included the darkgray module, the darkturquoise module and the greenyellow module (Figure 4B). The greenyellow module identified 452 genes that exhibited opposite expression patterns between the R and S-lines at any sampling time point. The darkturquoise module, with 487 identified genes, was highly associated with DH40R at 8 dai. The darkgray module (758 genes) was highly associated with 8 dai of DH199S and 5, 8, and 13 dai of DH40R (Supplementary Table 6). According to the results of morphological and histological analyses, DH40R exhibited no galls at 8 dai, while DH199S exhibited galls at that time point. In addition, the cortical infection rate of DH40R remained basically unchanged from 8 dai onward. Thus, the genes in these three modules played vital roles in the transition of Chinese cabbage from normal growth to gall appearance in the roots and are worthy of further analysis. The results of KEGG analysis for the greenyellow module showed that the significant mapped pathways included “plant hormone signal transduction” and “metabolism” (Supplementary Figure 2). The genes in the darkturquoise module were mainly associated with the significant mapped pathways including “environmental adaptation” and “organismal systems” (Supplementary Figure 2). The genes in the darkgray module were mainly associated with significant mapped pathways included “biosynthesis of other secondary metabolites” and “phenylpropanoid biosynthesis” (Supplementary Figure 2). The above genes in these modules may play important roles in Chinese cabbage seedling responses to *P. brassicae* to ensure normal early growth and morphogenesis after infection by *P. brassicae*.

### Key Genes Involved in Resistance to *P. brassicae* Screened via WGCNA

**FIGURE 5:**
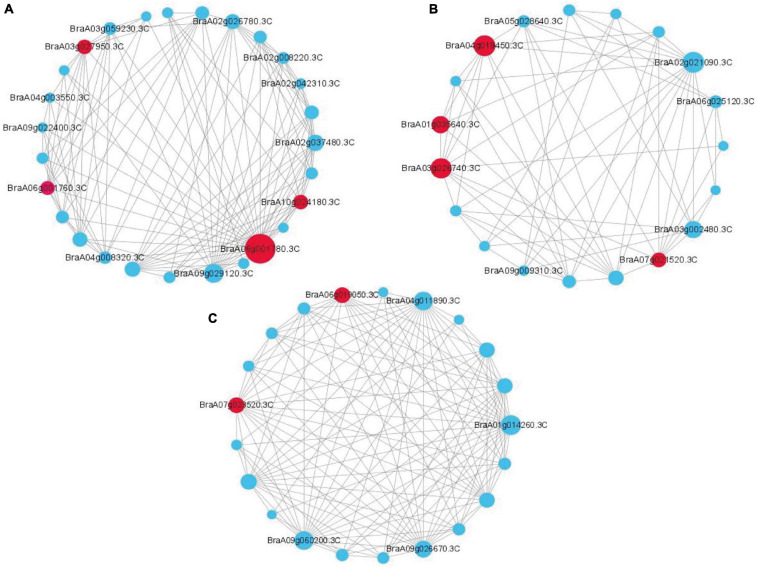
Coexpression network analysis of three key modules. **(A)** The correlation networks in the darkgray module. **(B)** The correlation networks in the darkturquoise module. **(C)** The correlation networks in the greenyellow module. Candidate hub genes are shown in red.

WGCNA can also be used to construct gene networks, in which each node represents a gene and the connecting lines (edges) between genes represent coexpression correlations (Langfelder and Horvath, 2008). We screened the top 150 genes from different modules to establish a network according to module membership (kME). Each node represents a gene, and the connecting lines (edges) between genes represent gene coexpression correlations. The significantly coexpressed genes were selected and ranked by node size, and the three key modules were visualized. Removing unknown genes, ten genes were highlighted after WGCNA and interaction network analyses, including *BraA06g001780.3C* (*probable 2-oxoglutarate-dependent dioxygenase AOP1*, *AOP1*), *BraA03g027950.3C* (*lipid transfer protein EARLI 1-like*, *EARLI*), *BraA10g024180.3C* (*Brassica rapa peroxidase 56, POD56*), *BraA06g001760.3C* (*probable 2-oxoglutarate-dependent dioxygenase AOP1*, *AOP1*), *BraA04g019450.3C* (*NDR1/HIN1-like protein 13, NHL13*), *BraA02g021090.3C* (*lipoxygenase 4, LOX4*), *BraA03g002480.3C* (*NDR1/HIN1-like protein 3, NHL3*), *BraA07g021520.3C* (*NAC transcription factor 29-like, NAC29*), *BraA06g019050.3C* (*auxin response factor 2-like, ARF2*) and *BraA07g023520.3C* (serine/threonine-protein kinase EDR1-like, EDR1); these genes were considered to be the hub genes for resistance to *P. brassicae* in Chinese cabbage (Figures 5A–C).

### Quantitative RT-PCR Validation

**FIGURE 6:**
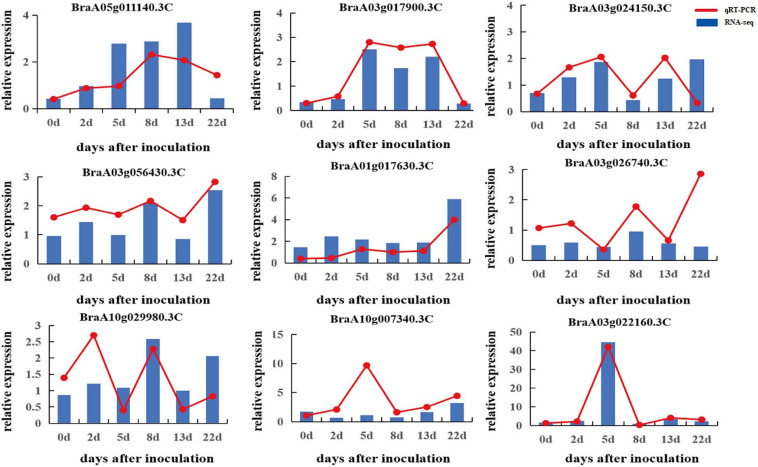
Validation of RNA-seq data by qRT-PCR.

To verify the quality of the RNA-seq and differential expression level data, nine DEGs were selected from 5,750 rDEGs to perform quantitative RT-PCR (Figure 6). These genes included two genes encoding an auxin-repressed 12.5 kDa protein, one gene involved in the JA signaling pathway, two genes involved in signal transduction, two genes from the darkturquoise module and two genes from the darkgray module. The changes in the expression of selected genes according to qRT-PCR showed a similar expression tendency to the RNA-seq data, which indicated that the transcriptomic profiling data were reliable.

## Discussion

In this work, we provide a comparative cytological transcriptome analysis of two *B. rapa* lines (the clubroot-resistant line DH40R and the clubroot-susceptible line DH199S) after *P. brassicae* infection to highlight the complex regulatory network of clubroot resistance in Chinese cabbage.

### Both Primary and Secondary Infection Occurred in Clubroot-Resistant and Clubroot-Susceptible Plants

The primary zoospores of *P. brassicae* are released from the resting spores to infect root hairs and then divide to form secondary sporangia. This complex process is the life cycle of *P. brassicae* (Kageyama and Asano, 2009). However, it is not clear at which stage and how resistance protects against *P. brassicae* in Chinese cabbage. In our study, microscopy observations revealed that root hair infection (2 dai) and cortical infection (5 dai) were both present in DH199S and DH40R and that the infection rate of the DH199S cortex began to increase significantly after 8 dai, while the cortex in DH40R remained basically unchanged.

The above cytological observations showed that disease-resistant DH40R could not completely resist *P. brassicae* infection and that its root hairs and cortex could be infected; however, *P. brassicae* could not grow and multiply indefinitely in its cortex cells. The disease-susceptible line DH199S could not resist the growth and reproduction of *P. brassicae* and eventually developed galls on its roots. This finding was not in agreement with a previous study (Chen et al., 2016), in which secondary infection did not occur in clubroot-resistant lines. This work was also not in agreement with a previous study in which gall development was visible in the clubroot-resistant genotype at 21 dai (Du et al., 2017).

### rDEGs May Regulate Clubroot Disease in Chinese Cabbage

In this study, there were no uninoculated controls at each time point, therefore, we proposed the concept of rDEGs when a gene was identified as a DEG between the R- and S-lines, but also identified as a DEG in the R-line compared with the R-previous period line at the corresponding time points. It was more convinced and targeted to discover the pivotal genes that lead to the difference between the R- and S- lines. A total of 5,750 rDEGs were detected in DH40R vs. DH199S at the primary and secondary infection stages. Among these rDEGs, some important genes related to plant hormone signal transduction, plant-pathogen interactions, disease resistance proteins and transcription factors were investigated. In addition, 10 hub genes interacting with rDEGs were revealed by WGCNA and are discussed below.

### Auxin Gene Family Responses to *P. brassicae* Infection

Plant hormones play key signaling roles in host-*P. brassicae* interactions, and many auxin-activated and auxin-repressed genes are well known for their pivotal roles in plant development and defense responses (Lee et al., 2012). Auxin has been associated with cell elongation/expansion and is induced at the *P. brassicae* primary infection stage, and the accumulation of auxin leads to gall formation in roots (Ludwig-Müller, 2014). Out of 5,750 rDEGs, we screened 67 auxin-related genes, including 23 and 26 genes belonging to the Aux/IAA and small auxin up RNA (SAUR) families, respectively. Four, eleven, and three genes belonged to the auxin response factor (ARF), Gretchen Hagen 3 (GH3) and auxin-repressed protein (ARP) groups, respectively (Supplementary Table 7). SAUR proteins act as key regulators of auxin synthesis and transport, and SAUR upregulation could be helpful to decrease the IAA content and relieve the enlargement and expansion of root cells in clubroot-resistant plants (Ning et al., 2019). The hormone auxin is a key signal for plant growth and development that acts through the ARF transcription factors, and the ARF family has thus far been shown to be involved in plant development and responses to biotic and abiotic stresses (Truskina et al., 2020). GH3 proteins are involved in various responses of plants to abiotic and biotic stresses (Park et al., 2007). One GH3 family member, *GH3.11* (*jasmonate resistant 1*, *JAR1*), catalyzes the formation of the isoleucine conjugate of JA, and the *JAR1* mutant increases susceptibility to clubroot disease (Agarwal et al., 2011). In *A. thaliana*, several members of the GH3 family are upregulated during clubroot infection (Siemens et al., 2006).

In our work, 33 auxin-related genes were found to be upregulated in the clubroot-resistant line DH40R vs. the clubroot-susceptible line DH199S at the primary infection stage (2 dai), and four *ARF* genes were all upregulated (Supplementary Table 7). In addition, six genes were found to be upregulated in the clubroot-resistant line DH40R vs. DH199S throughout the entire infection stage, such as the hub gene *BraA06g019050.3C* (*ARF2*), which was highly expressed at 0, 2, 5, 8, 13, and 22 dai in DH40R. These genes could play roles in attempts by the host plant to regain the regulatory control of auxin homeostasis and in increased resistance to clubroot disease. However, three auxin-related genes (*BraA01g042940.3C*, *BraA06g033000.3C* and *BraA04g032040.3C*) were upregulated in the susceptible line DH199S vs. the clubroot-resistant line DH40R at 0, 2, 5, 8, 13, and 22 dai, which may have increased the content of auxin and led to gall formation in the developing roots.

### Pathogenesis-Related Proteins Contribute to Defense Against Clubroot

Previous studies have shown that *PR* genes can confer enhanced resistance against both biotic and abiotic stresses (Gupta et al., 2013). In *B. napus*, three *PR* genes, *PR1*, *PR2*, and *PR4* were consistently upregulated in both host genotypes during secondary infection by *P. brassicae* (Galindo-Gonzalez et al., 2020). The expression of *PR1* family genes has long been used as a marker of salicylic acid (SA)-mediated disease resistance, and the upregulation of *PR1* proteins throughout the infection stage, combined with their localization to the apoplast, which is an important interface for plant-microbe interactions, means that they can be regarded as a potential antimicrobial function protein family (Breen et al., 2017). In this study, we identified six *PR1* genes, *BraA06g003490.3C* (*PR1*) was only continuously upregulated at 2 and 5 dai and downregulated at 8, 13, and 22 dai in DH40R vs. DH199S, while *BraA03g042600.3C* (*PR1*) was upregulated at 8 and 22 dai and downregulated at 5 dai in DH40R (Supplementary Table 7). We speculated that the same type of gene may play a role in disease resistance at different stages.

Chitin constitutes 25% of the cell wall of *P. brassicae* and is the main component of the cell wall in *P. brassicae* (Moxham and Buczacki, 1983). Chitinases are a category of pathogenesis-related proteins that are believed to function as guardians against chitin-containing pathogens, and chitinase genes are induced in clubroot-susceptible genotypes during *P. brassicae* infection (Chen Q. et al., 2018). There are also several reports showing that different types of chitinase function against different pathogens in different plant species (Chen et al., 2016). In this study, we identified 12 chitinase genes (two *CH25* and ten *CHB4*), among which two *CH25* were downregulated at 2 dai inDH40R and seven *CHB4* were upregulated at 2 dai in DH40R. In addition, one chitinase gene (*BraA03g018400.3C, CHB4*) continuously showed high expression in DH40R at 0, 2, 5 8, 13, and 22 dai. The results demonstrated that different chitinases may play distinct roles at different infection stages among different genotypes.

### Antioxidant Response to *P. brassicae* Infection

Various signals of higher antioxidant abilities are associated with the activation of ROS, which contributes to suppressing fungal growth and regulating host PCD and hypersensitive response (HR); therefore, the resistant genotype shows a higher antioxidant ability than susceptible rapeseed (Mei et al., 2019). In *B. napus*, compared to the resistant genotype “Laurentian,” the susceptible line “Brutor” seems to turn off genes related to ROS metabolism earlier (Galindo-Gonzalez et al., 2020). In our work, 49 peroxidase genes (*PODs*), 11 thioredoxin genes, and two catalase (*CAT*) and protein kinase (Pkinase) genes were identified (Supplementary Table 7). In *Brassica napus*, *POD* continuously increases defense against *P. brassicae* throughout the infection period in the resistant genotype ZHE-226 relative to the susceptible line (Mei et al., 2019). Most peroxidase genes began to be upregulated at 5 dai in DH40R vs. DH199S, downregulated at 13 dai and then maintained for a short time. The expression of the hub gene *BraA10g024180.3C* (*POD56*) in DH40R was upregulated at 5, 8, and 13 dai compared to that in DH199S. These findings are consistent with a previous report showing that DH40R initiates antioxidant reactions earlier and maintains them for a longer time than DH199S to maintain normal biological activities and plant health. These results indicated that higher antioxidant ability probably contributes to resistance to clubroot development in *B. rapa* and effectively reduces the damage to plants caused by *P. brassicae*.

### The *WRKY* and *MYB* Transcription Factors Regulation in *P. brassicae* Infection

Transcription factors play a key role in the initiation and regulation of gene transcription (Zhang and Feng, 2014; Irani et al., 2018). The *WRKY* and *MYB* transcription factor families play key roles in the defensive response against pathogen attacks (Eulgem and Somssich, 2007). WRKY proteins are a superfamily of transcription factors with up to 100 representatives in *Arabidopsis*, and family members appear to be involved in the regulation of various physiological programs, including pathogen defense, senescence and trichome development (Eulgem et al., 2000). In *Brassica juncea* var. *tumida* Tsen, *WRKY22s* have been shown to act as positive regulators of the *P. brassicae* resistance-enhanced genotype induced by the biocontrol strain *Zhihengliuella aestuarii* (Lai et al., 2019). Previous studies in plants have suggested that *AtWRKY28* and *AtWRKY75* are transcriptional regulators of SA and JA/ET-dependent defense signaling pathways in *Arabidopsis* (Chen et al., 2013).

MYB proteins function as transcription factors in regulatory networks controlling development, metabolism and responses to biotic and abiotic stresses (Dubos et al., 2010). *MYB15* is required for the defense-induced synthesis of G-rich lignin, which contributes to disease resistance against a hemibiotrophic bacterial pathogen (Chezem et al., 2017). The transcription factor *MYB44* is a member of the *MYB* family of transcription factors and is better studied in *Arabidopsis*. *AtMYB44* is a transcription factor that functions in association with the ethylene signaling pathway to regulate defense responses (Liu et al., 2011). *MYB44* is a negative regulator of ABA, stress, and wounding responses and blocks senescence; it consistently functions to maintain growth in the case of physical damage or stress (Jaradat et al., 2013). In addition, the overexpression of *SmMYB44* in eggplant increases resistance to *bacterial wilt* (Qiu et al., 2019).

In our study, we identified 57 genes related to *WRKY* TFs and 52 *MYB* genes (Supplementary Table 7), among which 11 *WRKY* TFs (*BraA03g028140.3C*, *BraA04g002600.3C, BraA04g028830.3C, BraA04g028840.3C, BraA05g001850.3C, BraA06g028240.3C, BraA08g012800.3C, BraA09g025880.3C, BraA09g026560.3C, BraA09g034360.3C*, and *BraA09g035200.3C*) were upregulated in DH40R vs. DH199S at all-time points after inoculation with *P. brassicae*. *MYB44* (*BraA09g057480.3C*), *MYB59* (*BraA07g008760.3C*), and *MYB86* (*BraA05g034360.3C*) were consistently highly expressed in DH40R at 0, 2, 5, 13, and 22 dai. These genes were inferred to be involved in the clubroot resistance-regulating process throughout all infection stages.

### Disease Resistance Proteins

Disease resistance proteins contain an NB-ARC (nucleotide-binding adaptor shared by APAF-1, R proteins, and CED-4) domain along with an LRR domain, and some also bear a toll interleukin 1 receptor (TIR) domain (Wen et al., 2017). In previous studies, *CRa* (Ueno et al., 2012), *CRb* (Hatakeyama et al., 2013), *Crr1a* (Hatakeyama et al., 2016) and *Rcr2* have been reported to belong to the NB-TIR-LRR family and have been considered candidate genes for several clubroot resistance loci in *B. rapa*. Among the 5,750 examined rDEGs, 28 disease resistance genes (Supplementary Table 7) containing a TIR domain showed significantly higher expression in DH40R vs. DH199S at least in one time period; notably, most of these genes were upregulated in DH40R vs. DH199S at 8 dai and 22 dai, while one gene (*BraA01g021410.3C*) was only upregulated in DH40R vs. DH199S at 2 dai. Overall, the available results indicate that the upregulated resistance genes in DH40R could be induced and achieve a more robust resistance response at the secondary infection stage than in the primary infection stage.

### The Other Key Genes Involved in Resistance to *P. brassicae*

In this work, the remaining eight hub genes from three modules were obtained through WGCNA and were also considered worthy of attention in the following analyses. *ENHANCED DISEASE RESISTANCE 1 (EDR1)* encodes a *CTR1*-like kinase and was previously reported to prevent the inappropriate initiation of cell death, growth inhibition, and senescence (Frye et al., 2001; Tang et al., 2005). The expression level of *BraA07g023520.3C(EDR1)* was significantly higher in DH40R vs. DH199S during the entire infection stage. *NAC* transcription factors (TFs) play vital roles in plant development, biotic and abiotic responses and hormone signaling, *Arabidopsis* ATAF1 and ATAF2 are negative regulators of defense responses against bacterial and fungal pathogens (Delessert et al., 2005; Wang et al., 2009). In this study, the hub gene *BraA06g025120.3C* (*NAC29*) was upregulated in DH40R relative to DH199S at 2, 8, 13, and 22 dai. The results demonstrated that these two hub genes may play key roles in regulating clubroot resistance reactions at the primary and secondary infection stages.

Fatty acid desaturation is an important part of the plant defense reaction and *LOXs* encoding lipoxygenase, which catalyze the oxygenation of fatty acids *LOX4* (*lipoxygenase 4*) expressed in phloem-associated cells plays a key role in controlling plant defense against nematode infection (Ozalvo et al., 2014; Chauvin et al., 2016). The expression levels of the hub gene *BraA02g021090.3C* (*LOX4*) at 8, 13, and 22 dai in DH40R were significantly higher than those in DH199S. *NDR1/HIN1-like (NHL)* genes play crucial roles in pathogen-induced plant responses to biotic stress (Blazquez et al., 2016). *NHL13* is required for plant immunity, as *NHL13* mutant plants display enhanced disease susceptibility (Xin et al., 2015). The expression levels of *BraA03g002480.3C(NHL3)* and *BraA04g019450.3* (*NHL13*) at 8 dai in DH40R were significantly higher than those in DH199S. Tandem 2-oxoglutarate-dependent dioxygenases (*AOPs*) control glucosinolate biosynthesis in *Arabidopsis*, and glucosinolate is required for plant defense. In our study, the expressions of *BraA06g001760.3C* and *BraA06g001780.3C* (*AOP1*) at 5, 13, and 22 dai were found to be highly upregulated in DH40R relative to DH199S. Lipid metabolism indirectly involves or influences plant immunity, which might affect the energy flux from pathway to pathway or act as a physical barrier to impact the plant disease resistance response (Li et al., 2020). The expression level of *BraA03g027950.3C(lipid transfer protein EARLI 1)* was upregulated in DH40R vs. DH199S at 5, 13, and 22 dai. The results demonstrated that these six hub genes may play key roles in regulating clubroot resistance reactions at the secondary infection stage.

## Conclusion

**FIGURE 7:**
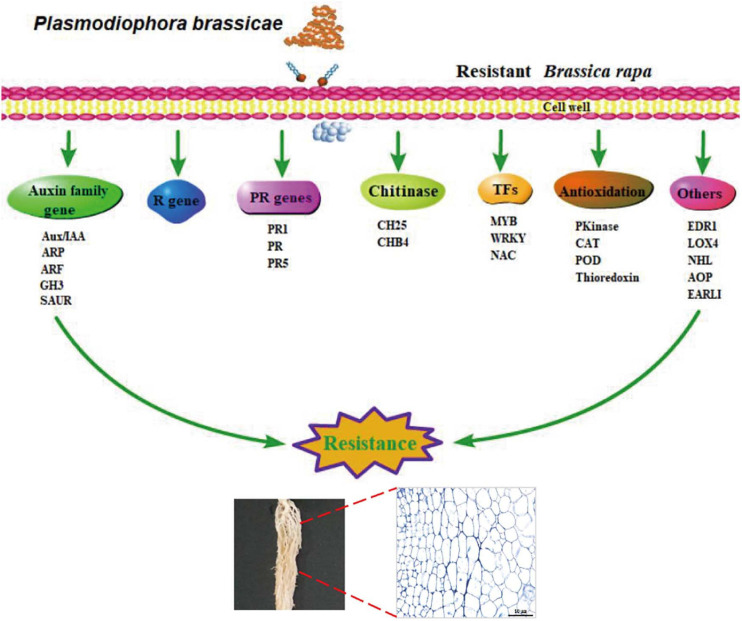
A simplified schematic diagram of the components of the resistance response to clubroot at transcriptional level.

In this study, we analyzed cytological and transcriptome profiles to investigate global transcriptome changes in clubroot-resistant and clubroot-susceptible Chinese cabbage at the primary and secondary stages of *P. brassicae* infection. Our results indicated distinct complex responses to *P. brassicae* at different infection stages between the different genotypes. In DH40R, the primary and secondary infection stages started at 2 and 5 dai, respectively, but with the extension of the infection time, cortical infection was blocked to prevent further reproduction of *P. brassicae* and disease development. Combined with transcriptome analysis, WGCNA and network analysis were used to reveal genes related to clubroot resistance. Specifically, genes associated with auxin, PR proteins, disease resistance proteins, oxidative stress, and *WRKY* and *MYB* transcription factors play important roles in clubroot resistance. Based on the above pathways and genes regulating clubroot resistance reactions, we proposed a simplified schematic diagram to explore the reactions of defensive resistance to clubroot at the transcriptional level (Figure 7). In particular, *ARF2*, *EDR1*, *LOX4*, *NHL3*, *NHL13*, *NAC29*, *AOP1*, *EARLI1*, and *POD56* may be worthy of more attention in future studies. In summary, our results could deepen the understanding of the molecular basis underlying the resistance of Chinese cabbage to clubroot.

## Data Availability Statement

The datasets presented in this study can be found in online repositories. The names of the repository/repositories and accession number(s) can be found in the article/Supplementary Material.

## Author Contributions

YY, XZ, and BT conceived the study. YY, SY, and XW designed the experiments. LQ, SY, and HS performed the experiments and analyzed the data. ZW, YZ, LL, and HL participated in the qRT-PCR and phenotype identification. LQ and HS drafted the manuscript. YY and HS revised the manuscript. All the authors have read and approved the publication of the manuscript.

## Conflict of Interest

The authors declare that the research was conducted in the absence of any commercial or financial relationships that could be construed as a potential conflict of interest.

## Publisher’s Note

All claims expressed in this article are solely those of the authors and do not necessarily represent those of their affiliated organizations, or those of the publisher, the editors and the reviewers. Any product that may be evaluated in this article, or claim that may be made by its manufacturer, is not guaranteed or endorsed by the publisher.

## Footnotes

1
http://brassicadb.org/brad/datasets/pub/Genomes/Brassica_rapa/V3.0/

2
https://www.takarabiomed.com.cn

## Supplementary Material

Supplementary Figure 1 Infection dynamics in the root hairs of DH40R and DH199S. **(A–D)** Segments of control roots (0 dai). **(E–H)** Segments of inoculated roots at 2 dai. **(I,J)** Segments of inoculated roots at 5 dai. **(K,L)** Segments of inoculated roots at 8 dai.

Supplementary Figure 2 Kyoto Encyclopedia of Genes and Genomes (KEGG) enrichment analysis of three modules.

Supplementary Table 1 The FPKM and annotation of all expressed genes.

Supplementary Table 2 The information of the primers used in qRT-PCR.

Supplementary Table 3 The disease index, root hair and cortical infection rates of DH40R and DH199S.

Supplementary Table 4 Sample description and mapping rate for samples.

Supplementary Table 5 The FPKM and annotation of rDEGs.

Supplementary Table 6 The module membership and gene significance of genes in three modules of WGCNA.

Supplementary Table 7 The FPKM and annotation of auxin-related genes, pathogenesis-related genes, *MYB*s, *WRKY*s, disease resistance proteins, antioxidation genes, and ten hub genes involving clubroot resistances.
