# Supplementary figures and images for: Transcriptome and Coexpression Network Analyses Reveal Hub Genes in Chinese Cabbage (Brassica rapa L. ssp. pekinensis) During Different Stages of Plasmodiophora brassicae Infection
Source: Front Plant Sci. 2021 Aug 10;12:650252. doi: 10.3389/fpls.2021.650252 (PMC8383047; doi:10.3389/fpls.2021.650252)

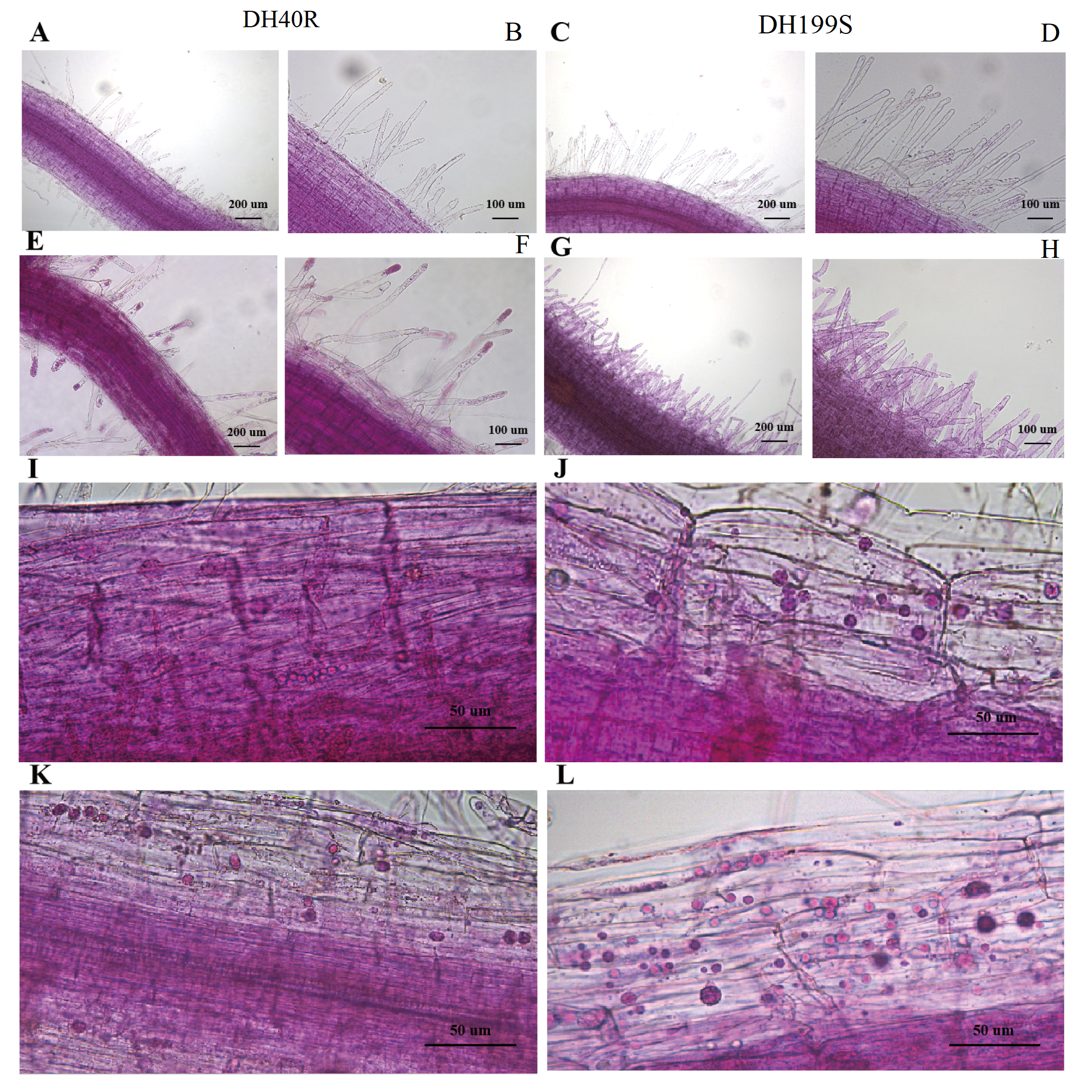

Supplement: Supplementary Figure 1 — Infection dynamics in the root hairs of DH40R and DH199S. (A–D) Segments of control roots (0 dai). (E–H) Segments of inoculated roots at 2 dai. (I,J) Segments of inoculated roots at 5 dai. (K,L) Segments of inoculated roots at 8 dai. [file Image_1.TIF]

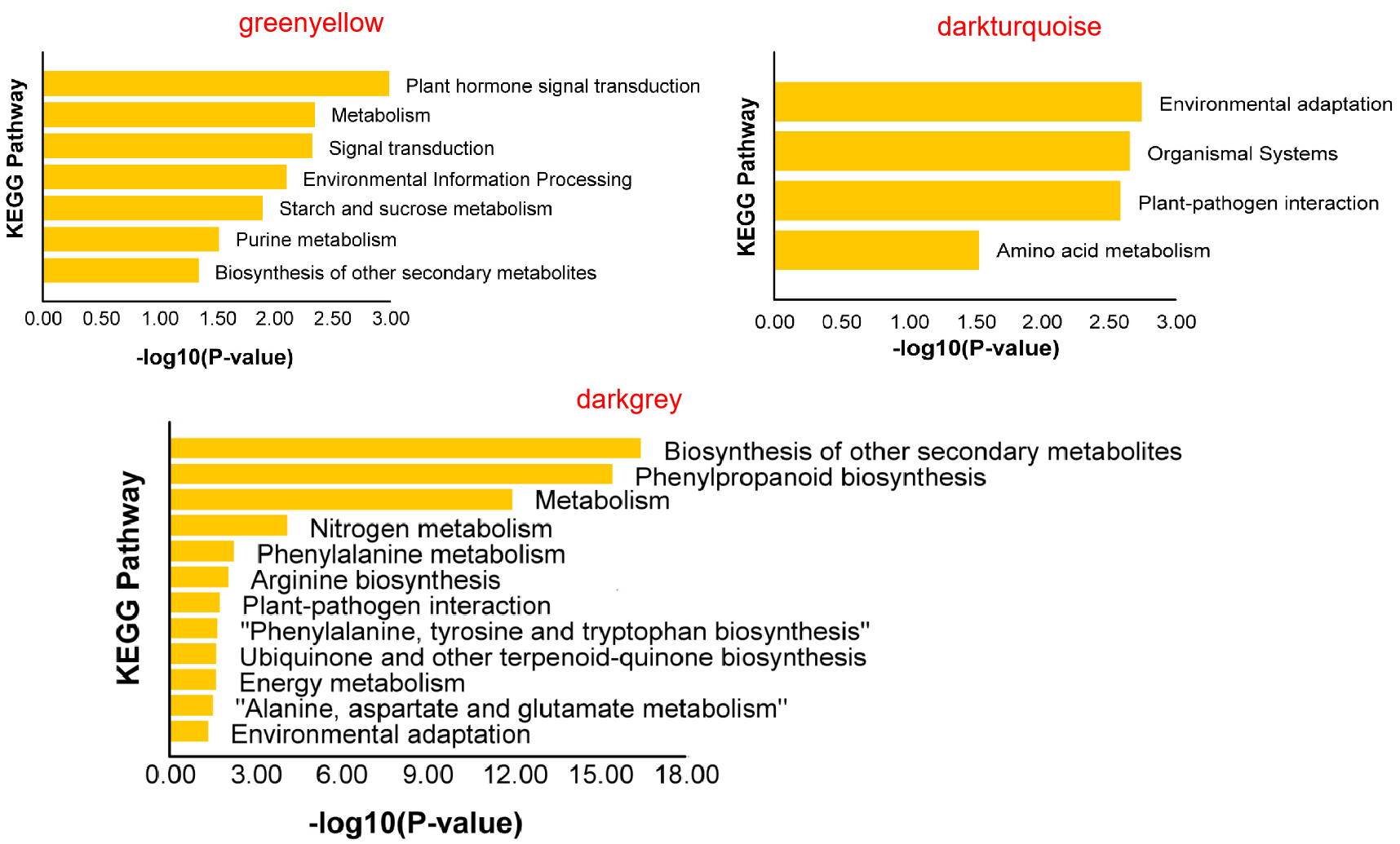

Supplement: Supplementary Figure 2 — Kyoto Encyclopedia of Genes and Genomes (KEGG) enrichment analysis of three modules. [file Image_2.TIF]
